# Phosphoproteomic analysis of the non-seed vascular plant model *Selaginella moellendorffii*

**DOI:** 10.1186/1477-5956-12-16

**Published:** 2014-03-17

**Authors:** Xi Chen, Wai Lung Chan, Fu-Yuan Zhu, Clive Lo

**Affiliations:** 1 School of Biological Sciences, The University of Hong Kong, Pokfulam Hong Kong, China; 2 Wuhan Institute of Biotechnology, Wuhan, Hubei, China

**Keywords:** Selaginella, IMAC, PEG fractionation, Phosphosites, Phosphorylation motif

## Abstract

**Background:**

Selaginella (*Selaginella moellendorffii*) is a lycophyte which diverged from other vascular plants approximately 410 million years ago. As the first reported non-seed vascular plant genome, Selaginella genome data allow comparative analysis of genetic changes that may be associated with land plant evolution. Proteomics investigations on this lycophyte model have not been extensively reported. Phosphorylation represents the most common post-translational modifications and it is a ubiquitous regulatory mechanism controlling the functional expression of proteins inside living organisms.

**Results:**

In this study, polyethylene glycol fractionation and immobilized metal ion affinity chromatography were employed to isolate phosphopeptides from wild-growing Selaginella. Using liquid chromatography-tandem mass spectrometry analysis, 1593 unique phosphopeptides spanning 1104 non-redundant phosphosites with confirmed localization on 716 phosphoproteins were identified. Analysis of the Selaginella dataset revealed features that are consistent with other plant phosphoproteomes, such as the relative proportions of phosphorylated Ser, Thr, and Tyr residues, the highest occurrence of phosphosites in the C-terminal regions of proteins, and the localization of phosphorylation events outside protein domains. In addition, a total of 97 highly conserved phosphosites in evolutionary conserved proteins were identified, indicating the conservation of phosphorylation-dependent regulatory mechanisms in phylogenetically distinct plant species. On the other hand, close examination of proteins involved in photosynthesis revealed phosphorylation events which may be unique to Selaginella evolution. Furthermore, phosphorylation motif analyses identified Pro-directed, acidic, and basic signatures which are recognized by typical protein kinases in plants. A group of Selaginella-specific phosphoproteins were found to be enriched in the Pro-directed motif class.

**Conclusions:**

Our work provides the first large-scale atlas of phosphoproteins in Selaginella which occupies a unique position in the evolution of terrestrial plants. Future research into the functional roles of Selaginella-specific phosphorylation events in photosynthesis and other processes may offer insight into the molecular mechanisms leading to the distinct evolution of lycophytes.

## Background

Selaginella (*Selaginella moellendorffii*) is a lycophyte believed to be originated from the earliest vascular plants approximately 410 million years ago [1]. Although lycophytes have existed twice as long as angiosperms, they have not evolved flowers and seeds since their divergence from other plant lineages. For this reason, Selaginella has been selected as a model plant to understand the early evolution of developmental and metabolic processes that are unique to vascular plants [2]. After a bacterial artificial chromosome library was constructed from clonally propagated plants [3], the complete Selaginella genome sequence was released in 2007 [4]. Subsequently, a number of investigations on Selaginella were launched in different areas including gene evolution [5-10], pathway conservation [11-16], genomic DNA composition and methylation [17-19], sRNA functions and RNA editing [20,21], and transposons [22]. Interestingly, Selaginella was found to utilize genes significantly different from flowering plants to generate secondary metabolites with potentials for pharmaceutical applications [23-26]. Meanwhile, proteomic investigations on this non-seed vascular plant model have not been extensively reported. A two-dimensional electrophoresis-based approach was recently employed to explore the desiccation tolerance mechanism in the resurrection plant *Selaginella tamariscina*[27].

Post-translational modifications (PTMs) play important roles in the regulation of protein functions and they occur at distinct amino acid side chains or peptide linkages. It has been estimated that more than 200 types of PTMs exist in proteins [28]. Protein phosphorylation, principally on serine, threonine or tyrosine residues, is one of the most important and well-investigated PTMs. It represents a reversible molecular switch controlled by protein kinases and protein phosphatases, either activating or inactivating the target proteins [29]. Approximately one-third of all proteins in eukaryotic cells were estimated to be phosphorylated at any given time [30]. In plants, protein phosphorylation plays a central role in virtually all cellular processes, including carbon and nitrogen metabolism, growth and development, transcription and translation, responses to abiotic and biotic stresses, cell cycle, and apoptosis [31]. Therefore, the identification of protein kinases and phosphatases, their substrates, and the phosphorylation sites involved is crucial for the understanding of many fundamental processes in plants. Interestingly, Arabidopsis contains over 1000 protein kinases [32], which is twice as many as those in human, while the two genomes share similar number of genes [33]. Hence, protein phosphorylation events in plants appear to be very different and more complicated than those in mammals. In fact, a number of plant protein kinases implicated in early events of signal transduction are unique with no mammalian orthologs [34].

Phosphoproteomic investigations in plants were initiated in recent years following the completion of different genome sequencing projects. The highly abundant ribulose-1,5-bisphosphate carboxylase/oxygenase (RUBISCO) protein, which accounts of about 50% of total soluble proteins [35], hindered the detection of low-abundant proteins including many phosphoproteins [36]. Polyethylene glycol (PEG) fractionation has been used as a cost-effective and contaminant-free procedure to remove RUBISCO for improved detection of low-abundant proteins [37-39]. In addition, phosphopeptide enrichment procedures, such as immobilized metal ion affinity chromatography (IMAC), are necessary to reduce the complexity of proteolyzed lysates for mass spectrometry analysis. IMAC is based on affinity purification through metal complexation with the phosphate group in phosphopeptides [40] and it has been adopted in Phosphoproteomic analysis in different plant systems [41-46].

In the present study, we used the PEG fractionation approach followed by the IMAC procedure to prepare Selaginella samples for phosphoproteome profiling and identified 1588 unique phosphorylation sites. Our dataset revealed features that are consistent with the Arabidopsis phosphoproteome. We further identified phosphorylation events that are conserved between Selaginella and angiosperm orthologous sequences. Novel and unique phosphosites were detected in several photosynthesis-related proteins in Selaginella. Phosphorylation motifs recognized by known protein kinase classes were revealed for both evolutionarily conserved and Selaginella-specific proteins.

## Results and discussion

### General features of the Selaginella phosphoproteome dataset

We employed the procedures of PEG fractionation (Additional file 1: Figure S1) and IMAC enrichment to isolate phosphopeptides from wild-growing Selaginella (*Selaginella moellendorffii*) for LC-MS/MS analysis. A total of 1593 unique phosphopeptides containing 1588 non-redundant phosphosites were discovered in our study (Additional file 2: Table S1). Among them, 1104 were identified with high confidence of localization (localization probability ≥ 95%), 116 with median confidence of localization (80% ≤ localization probability < 95%), and 368 with low confidence of localization (localization probability < 80%). Phosphosites with high confidence of localization were categorized into pSer (86.2%), pThr (13.3%), and pTyr (0.5%). The relative distribution of the three phosphorylated residues is consistent with previous reports for different flowering plant species [45,46]. As Ser/Thr kinases are commonly encoded in plant genomes, more frequent Ser and Thr phosphorylation events are expected. On the other hand, while typical Tyr-specific kinases are absent in plant genomes, a few plant kinases with dual specificity are believed to phosphorylate Tyr residues in proteins [47].

The 1104 confirmed phosphosites correspond to a total of 716 Selaginella proteins, 665 of them can be assigned to orthologous protein groups (Additional file 3: Table S2) using the OrthoMCL algorithm with a cut-off of E-5 e-value and 50% sequence match [48,49]. Forty two proteins are considered Selaginella-specific proteins since they could not be assigned to any OrthoMCL groups or do not have any matching sequences in the OrthoMCL database. These proteins may have evolved in lycophytes after their separation from other vascular plants including ferns and seed plants.

### Analysis of phosphosite locations in Selaginella proteins

**Figure 1:**
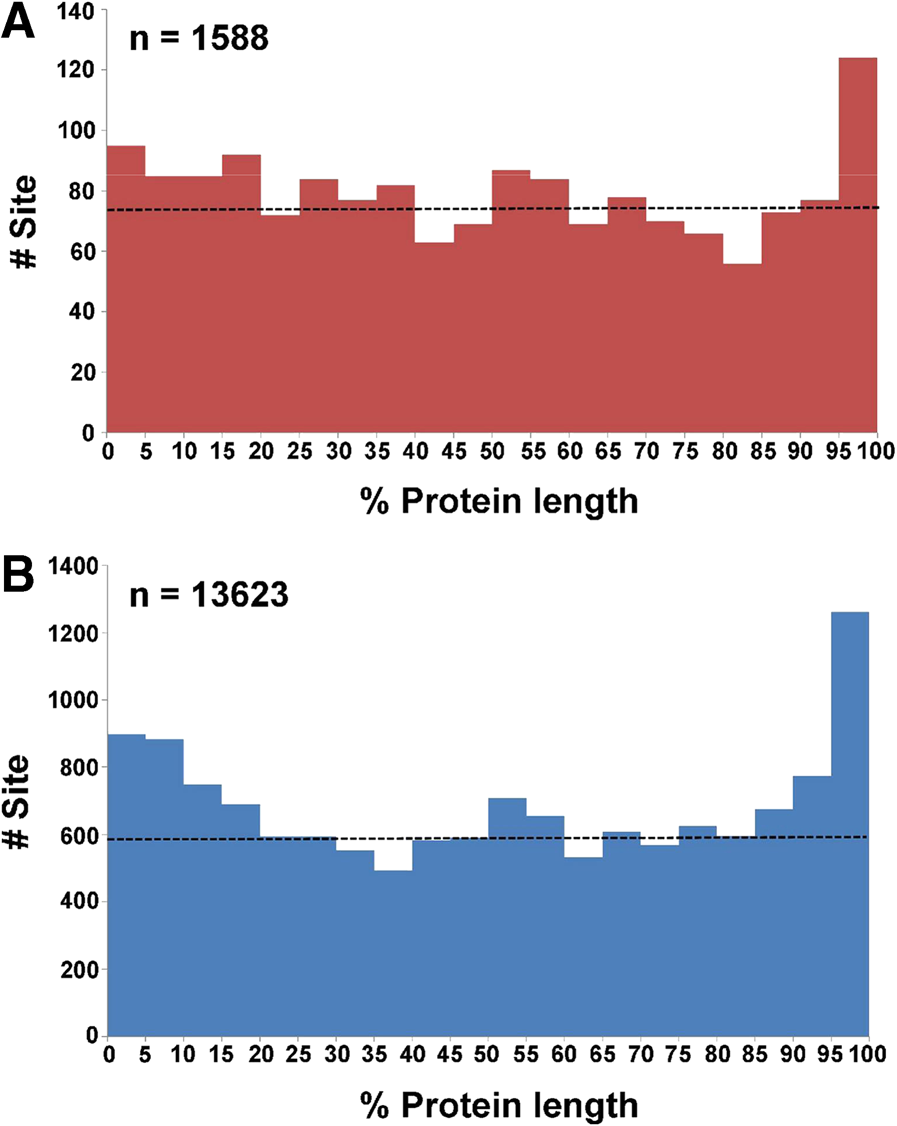
**The distribution of phosphorylation sites along the phosphoproteins of (A) Selaginella and (B) Arabidopsis.** The Arabidopsis dataset was retrieved from P^3^DB.

To analyze the locations of the identified phosphorylation sites, protein sequences were divided into 5% fractions and the number of phosphorylation events was counted within each fraction. As shown in Figure 1A, the highest number of phosphosites is found in the last fraction, i.e. the C-termini of proteins. We performed parallel analysis using an Arabidopsis phosphoproteome dataset (retrieved from P^3^DB) and found a very similar distribution pattern for the phosphosites (Figure 1B). Such phenomenon was also described in a phosphoproteomic study of mouse liver [50]. Hence, the more frequent C-terminal phosphorylation in proteins appears to be a common feature in different organisms, including plants and animals. The C-terminal region was suggested to be more exposed and flexible for protein phosphorylation [50].

### Functional categorization of the identified Selaginella phosphoproteins

**Figure 2:**
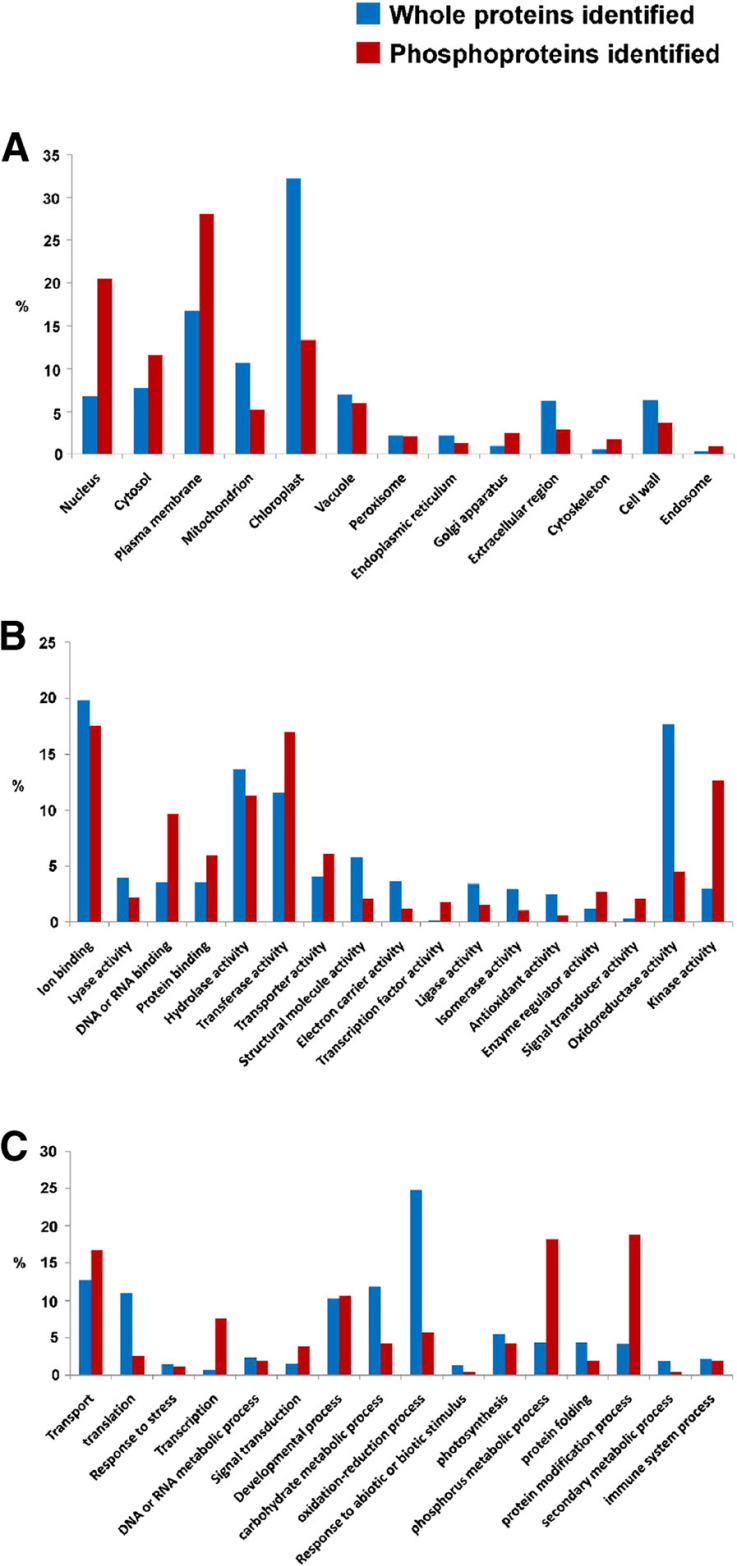
The GO annotation distribution of Selaginella whole proteins (before IMAC enrichment) and phosphoproteins in the categories of (A) cellular component, (B) molecular function and (C) biological processes.

To understand the functional distribution of the unique Selaginella phosphoproteins identified in this study, their cellular localization, molecular function, and biological processes were analyzed and compared with those of 2400 Selaginella proteins identified after LC-MS/MS analysis of PEG-fractionated samples without the IMAC enrichment procedure. Based on the comparison of Gene Ontology (GO) term annotations (Figure 2), the 3 most over-represented categories for the identified phosphoproteins in each GO vocabulary are: nucleus, plasma membrane and cytosol for “cellular component”; DNA/RNA binding, kinase activity, and transferase activity for “molecular function”; protein modification, phosphorus metabolic process, and transcription for “biological process”.

### Location of phosphosites in characterized protein domains

To determine whether the Selaginella phosphosites are located in known structural and/or functional protein domains, Pfam database search (Wellcome Trust Sanger Institute) was performed to extract domain information of our identified phosphoproteins. A total of 594 proteins with domain information were retrieved. Among the 893 phosphosites in these proteins, only 201 (22.5%) were located inside protein domains (Table 1). Our findings are consistent with the observations from Arabidopsis phosphoproteome analysis suggesting that phosphorylation events may not have significant impact on domain-associated functions [51,52].

**Table 1:** Location of phosphorylation sites on characterized protein domains

**Number of proteins possessing Pfam domain**		**Number of phosphorylation sites**^**(1)**^		
		**Pfam domain**^**(2)**^		**Total (%)**
		**Inside (%)**	**Outside (%)**	
pS	538	163 (21.0%)	612 (79.0%)	775 (100%)
pT	102	36 (31.6%)	78 (68.4%)	114 (100%)
pY	4	2 (50%)	2 (50%)	4 (100%)
All	594	201 (22.5%)	692 (77.5%)	893 (100%)

^(1)^All analyzed phosphorylation sites were confirmed with the localization probability above 95% deduced from Ascore.

^(2)^Location of phosphorylation sites relative to the conserved domains annotated in the Pfam database.

### Phosphorylation motif analysis

**Figure 3:**
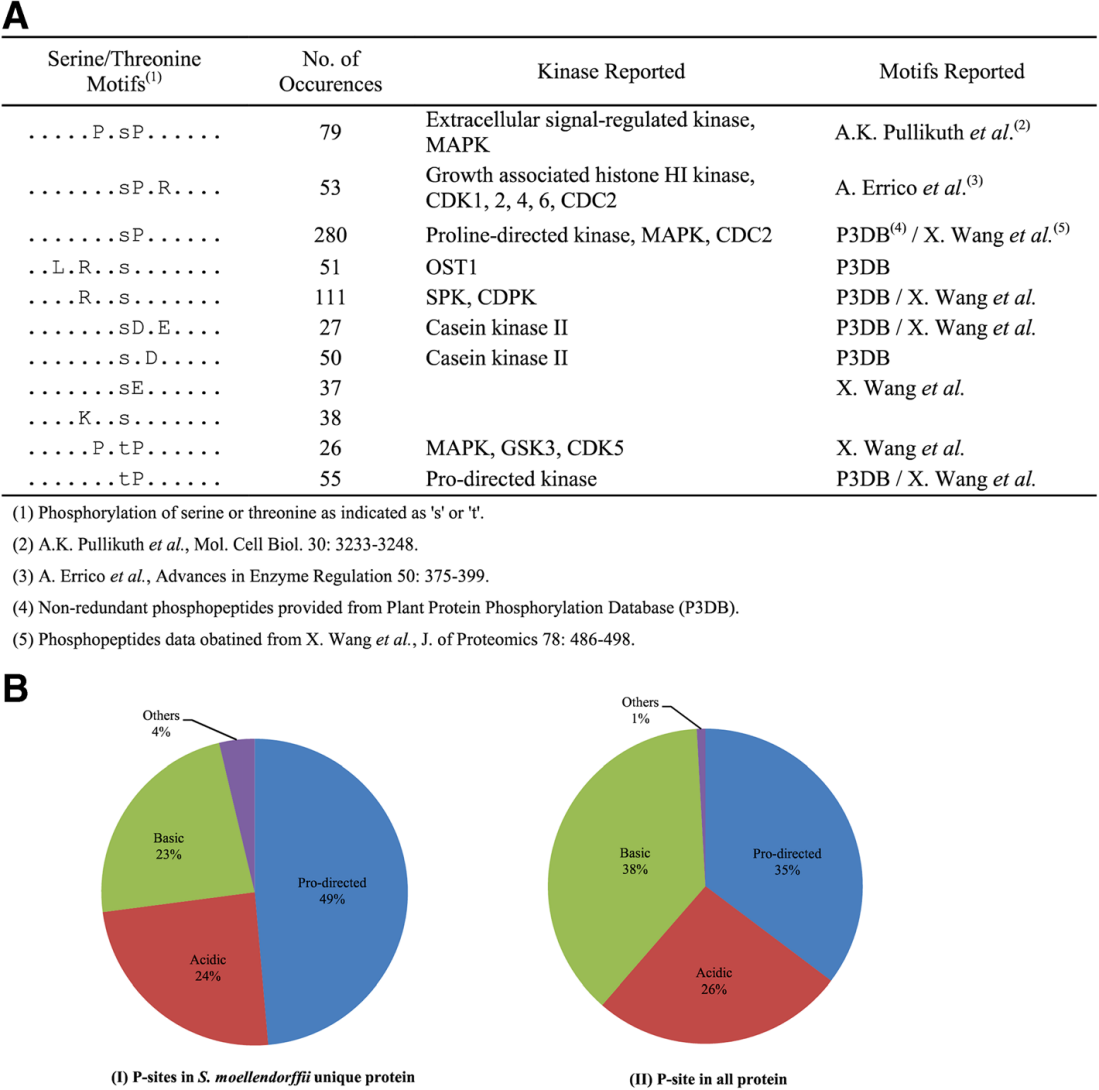
**Phosphorylation motifs in Sellaginella phosphoproteins. (A)** Motif-x analysis of phosphorylation peptides with localization probability 95%. **(B)** Motif classes distribution of Selaginella(Sm) specific phosphorylation site (P-site).

A phosphorylation motif search was performed on our phosphopeptide dataset (localization probability ≥ 95%) using the Motif-X algorithm [53]. Peptide sequences are aligned with their length adjusted to ±7 residues from the central phosphosite for data submission. Over-represented patterns of amino acid sequences were generated with a minimum occurrence of 20 and a significance value of 10^−6^. All together, we obtained a total of 11phosphorylation (9 Ser and 2 Thr) motifs containing at least one fixed amino acid aside from the central phosphorylated residue (Figure 3A). Both the Thr-motifs are Pro-targeted (TP and PXTP) and there are 3 Ser Pro-targeted motifs (SP, PXSP, SPXR). All these motifs are possible substrates of glycogen synthase kinase 3, cyclin-dependent kinase, and mitogen-activated protein kinase. In addition, 3 basophilic motifs (LXRXXS, RXXS, KXXS) likely to be associated with the activities of Ca^2+^-dependent protein kinase (CPK), Ca^2+^/calmodulin-dependent protein kinase, or protein kinase A were identified. Furthermore, 3 acidic motifs (SDXE, SXD, and SE) potentially recognized by casein kinase II were generated. We also performed parallel Motif-X analysis using Arabidopsis phosphopeptides retrieved from P^3^DB and those obtained by Wang et al. (2013). One of the Selaginella motifs, KXXS, was not generated from the Arabidopsis analysis. Thirty two (out of 38) occurrences of such motif correspond to proteins assigned with OrthoMCL group with e-value < E^−50^ (Additional file 4: Table S3), indicating that this basophilic motif is primarily associated with evolutionarily conserved proteins in Selaginella. Analysis of the 107 phosphosites in the Selaginella-specific proteins (those without any assigned OrthoMCL groups) revealed that they are more enriched in Pro-directed motifs when compared to all the identified phosphosites (49% vs 35%) (Figure 3B). On the other hand, the basophilic motifs are under-represented in the Selaginella-specific proteins when compared to all proteins identified (23% vs 38%). Consistently, a single SP motif with 36 occurrences was generated by Motif-X analysis for the 107 phosphosites found in the Selaginella-specific proteins. Taken together, most of the Selaginella phosphorylation events identified in this study are likely to be catalyzed by known classes of protein kinase classes in plants.

### Phosphorylation events in evolutionary conserved proteins

To identify phosphorylation events highly conserved between Selaginella and flowering plants, our identified phosphopeptides with confirmed phosphosites were clustered with phosphopeptides of Arabidopsis, rice, rapeseed, soybean and *Medicago truncatula* (retrieved from P^3^DB) by CD-HIT [54] using a sequence identity cutoff of 0.6 and an alignment bandwidth of 5 [44]. A total of 107 Selaginella phosphopeptides harboring 115 Ser/Thr phosphosites were found to cluster with phosphopeptides from the other plants. More than 80% (97/123) of those Selaginella phosphosites were found to have equivalent phosphosites in at least one other species. The majority (90/106) of the Selaginella proteins harboring the conserved phosphosites are evolutionarily conserved proteins belonging to Orthogroups identified with e values < E-50(Additional file 5: Table S4). Many of these proteins are involved in primary metabolism (e.g. Calvin cycle, glycolysis, TCA cycle, lipid biosynthesis), RNA processing, transcriptional regulation, cell cycle, protein phosphorylation (kinases), and signaling (e.g. G proteins, 14-3-3 protein, LRR-containing kinases). On the other hand, 17 of these highly conserved phosphorylation events are found in proteins with unknown functions. Selected phosphopeptide alignments containing highly conserved phosphosites in multiple plant species are shown in Table 2.

**Table 2:** Selected conserved phosphorylation sites within the orthologous proteins

**Accession**	**Phosphopeptides alignment**^**(1)**^	**Protein annotation**	**MapmanBin name**	**OrthoMCL group**	**E-value**
D8QZX4	VVLEGGsDDEGASTEAHGR	ILITYHIA	Not assigned.no ontology	OG5_128125	1E-181
AT1G64790.1	ALLEGGsDDEGASTEAQGR				
Os03g51140.1	AILEGGsDDEGASTEAHGR				
D8RKK4	SNsFVGTEEYIAPEIIK	KCBP-interacting protein kinase	Protein. stranslational modification	OG5_129514	1E-181
AT3G52890.2	SNsFVGTHEYLAPEIIK				
Os02g43740.1	SMsFVGTHEYLAPEIIK				
D8T2E8	ASGAFILTAsHNPGGPHEDFGIK	Phosphoglucomutase/phosphomannomutase family protein	Glycolysis.PGM	OG5_127226	1E-181
AT1G23190.1	ATGAFILTAsHNPGGPTEDFGIK				
Os03g50480.1	ATGAFILTAsHNPGGPTEDFGIK				
D8SQG3	sQAGTPEWMAPEVLR	Protein kinase superfamily protein	Signalling.MAP kinases	OG5_130251	1E-181
AT1G08720.1	sTAGTPEWMAPEVLR				
Os02g50970.2	sTAGTAEWMAPEVLR				
D8SCJ0	ALVANYNQtPR	Cell division cycle 5	RNA.regulation of transcription.MYB-related transcription factor family	OG5_128000	1E-181
AT1G09770.1	ALLANYSQtPR				
Os04g28090.1	ALLSSYSQtPR				
D8RHD1	GILAMDEsNATCGK	Aldolase superfamily protein	Glycolysis.aldolase	OG5_127143	1E-179
AT3G52930.1	GILAADEsTGTIGK				
Os05g33380.1	GILAADEsTGTIGK				
D8R365	GFVPILPGsPGSS	Na+/H + exchanger 1	Transport.unspecified cations	OG5_126729	1E-181
AT5G27150.1	GFVPFVPG*s*PTER				
Os11g42790.1	GFVPFVPGsPTER				
D8QX62	PsGSPPVPVMHsPPRPVTVK	Chromatin protein family	DNA.synthesis/chromatin structure	OG5_128357	1E-181
AT1G77180.1	AsGSPPVPVMHsPPRPVTVK				
Os02g52250.1	AsGSPPVPVMHsPPRPVTVK				
D8QU29	YHGHsMSDPGSTYR	Pyruvate dehydrogenase complex E1 alpha subunit	TCA/org.transformation.TCA. pyruvate DH.E1	OG5_127216	1E-176
AT1G59900.1	YHGH*s*MSDPGSTYR				
Medtr5g037700.1	YHGHsMSDPGSTYR				
D8SRX1	VVGTQAPVQLGsLR	GDP-D-mannose 3',5'-epimerase	Redox. ascorbate and glutathione.ascorbate. GME	OG5_135736	1E-181
AT5G28840.1	VVGTQAPVQLGsLR				
Os10g28200.1	VVSTQAPVQLGsLR				
D8RPK5	AHGPAVGLPTEDDMGNsEVGHNALGAGR	Phosphoglycerate mutase, 2,3-bisphosphoglycerate-independent	Glycolysis.phosphoglycerate mutase	OG5_129051	1E-181
AT1G09780.1	AHGTAVGLPSEDDMGNsEVGHNALGAGR				
Os05g40420.3	AHGTAVGLPSDDDMGNsEVGHNALGAGR				
D8SC69	VQsSSAIVVHPR	Receptor protein kinase TMK1 precursor, putative, expressed	--	OG5_141123	1E-181
Os03g50810.1	VQsPHAMVVHPR				
Glyma02g40980.1	VQsPNALVIHPR				
D8S0V4	QLsIDQFENEGR	BRI1 suppressor 1 (BSU1)-like 2	Protein.postranslational modification	OG5_132764	1E-181
AT1G08420.1	QLsIDQFENEGR				
Os12g42310.1	QLsIDQFENEGR				
D8QXA8	NFRPDsLLGEGGFGSVFK	Protein kinase superfamily protein	Protein.postranslational modification.kinase.receptor like cytoplasmatic kinase VII	OG5_147118	1E-163
AT1G07570.1	NFRPDsVLGEGGFGCVFK				
Glyma01g05160.1	NFRPDsLLGEGGFGYVYK				
D8RMG5	ALsPDRNDAFAMGDK	Splicing factor, putative	RNA.processing.splicing	OG5_127822	1E-181
AT5G64270.1	VLsPDRVDAFAMGDK				
Medtr2g009180.1	ILsPDRHDAFAAGEK				
D8RZ45	LTsFEALQSATK	ADP-glucose pyrophosphorylase family protein	Major CHO metabolism.synthesis.starch.AGPase	OG5_129964	1E-181
AT1G74910.1	RVsSFEALQPATR				
Os03g11050.3	RVsSFEALHSATK				
D8QZR7	AMKsPDPLEEQR	Protein kinase superfamily protein	Protein.postranslational modification.kinase	OG5_129183	1E-149
AT1G67580.1	MVKsPDPLEEQR				
Os02g39010.3	HMKsPDPLEEQR				
D8RF63	EIsDDEEEEEK	HEAT SHOCK PROTEIN 81.4	Stress.abiotic.heat	OG5_126623	1E-181
AT5G56000.1	EIsDDEEEEEK				
Os08g39140.1	EIsDDEDEEEK				
D8QX94	tSCGSPNYAAPEVISGK	SNF1-related protein kinase 1.3	Protein.postranslational modification	OG5_126655	1E-181
AT5G39440.1	tSCGSPNYAAPEVISGK				
Os03g17980.1	tSCGSPNYAAPEVISGK				
D8SX29	GEPNISyICSR	SHAGGY-related protein kinase dZeta	Protein.postranslational modification	OG5_126888	1E-181
AT2G30980.1	GEANISyICSR				
Os02g14130.1	GEANISyICSR				
D8R4T7	GGMTsHAAVVAR	Pyruvate orthophosphate dikinase	Glycolysis.PPFK	OG5_127082	1E-181
AT4G15530.3	GGMTsHAAVVAR				
Os05g33570.3	GGMTsHAAVVAR				
D8T8I0	GLDIDTIQQHYtV	H(+)-ATPase 1	transport.p- and v-ATPases.H + -exporting ATPase	OG5_127253	1E-181
AT2G18960.1	GLDIDTAGHHYtV				
Os04g56160.1	GLDIDTIQQNYtV				
D8R651	VHACVGGtDVR	Eukaryotic translation initiation factor 4A1	Protein.synthesis.initiation	OG5_126984	1E-181
AT3G13920.1	VHACVGGtSVR				
Medtr2g120800.1	VHACVGGtSVR				
D8QVP0	TIQFVDWCPtGFK	Tubulin alpha-3	Cell.organisation	OG5_126605	1E-181
AT5G19770.1	TVQFVDWCPtGFK				
Os03g51600.3	TIQFVDWCPtGFK				

^(1)^Phosphorylation sites are indicated as small letters.

Arabidopsis (AT); Rice (Os); Soybean (Glyma); Medicago trunculata (Medtr).

**Figure 4:**
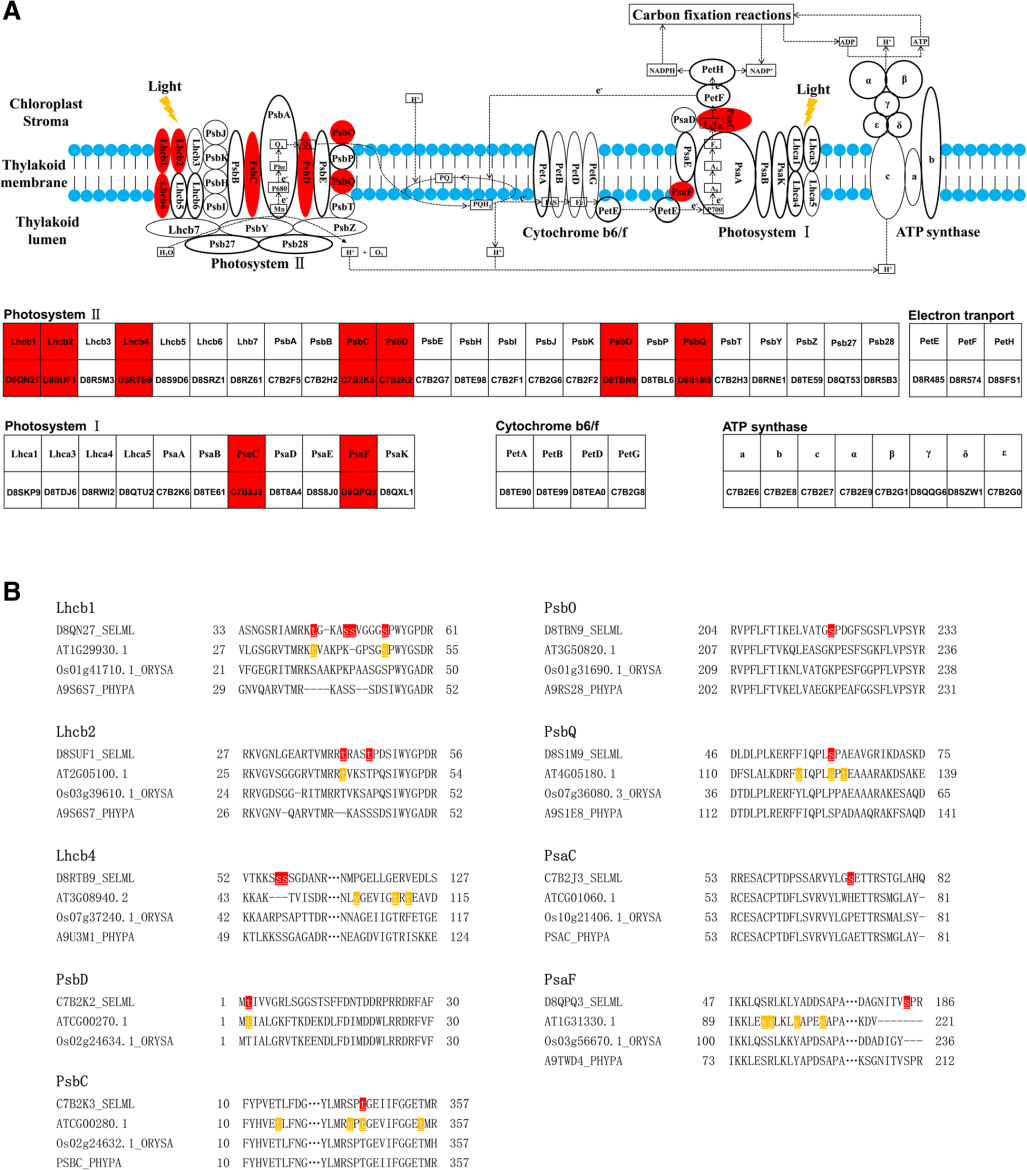
**Sellaginella phosphoproteins involved in photosynthesis. (A)** Graphical representation of the photosynthesis machineries using the KEGG classification system. Different proteins participating in light-dependent reactions are shown. Circles filled with red color denoted phosphoproteins with confirmed phosphosites identified in this study. UniProtKB accession numbers of Selaginella proteins are shown underneath the corresponding photosynthetic proteins. **(B)** Alignment of the identified Selaginella photosynthesis phosphoproteins (selected regions) with orthologous sequence from Arabidopsis, rice (ORYSA) and *P. patens* (PHYPA). Phosphosites identified in this study and in Arabidopsis are highlighted in red and yellow, respectively. Complete alignments of these proteins are available in Additional file 6: Figure S2. Phosphorylation information for the rice and *P. patens* sequences is not available.

Furthermore, we performed a close examination on the phosphorylation events in Selaginella photosynthesis-related proteins. The molecular machinery of photosynthesis has been highly conserved during plant evolution. Among our identified phosphoproteins with confirmed phosphosites, seven are involved in photosystem II (PSII) and two are involved in photosystem I (PSI) (Figure 4A). To reveal possible evolutionary significance, sequences were aligned with orthologs from Arabidopsis, rice, and *Physcomitrella patens* (moss), representing diverse lineages of dicot, monocot, and bryophytes, respectively (Figure 4B and Additional file 6: Figure S2). In all cases, phosphorylation information is only available for the Arabidopsis proteins. Sequences of rice and moss are included for examination of phosphorylatable residues at equivalent sites.

As the first link in the chain of light-dependent reactions, PSII captures photons and uses the energy to extract electrons from water molecules. The light-harvesting chlorophyll a/b protein complex LHCII in PSII is composed of three proteins, namely Lhcb1, Lhcb2, and Lhcb3 [55]. Non-phosphorylated LHCII functions as an antenna for PSII, but it migrates to come in contact with PSI following light-dependent phosphorylation of Lhcb1 and Lhcb2 [56] which is likely to occur at N-terminal Thr residues [57]. Although no explicit phosphosites were identified [55-57], STN7 kinase was strongly suggested to be required for phosphorylation of Lhcb1 and Lhcb2 to achieve state transitions between PSII and PSI [56]. In Selaginella, we detected N-terminal Thr phosphosites in D8QN27 (Lhcb1: Thr-44) and D8SUF1 (Lhcb2: Thr-42) (Figure 4B). Both phosphorylation events are conserved in Arabidopsis Lhcb1 and Lhcb2 [58,59]. Equivalent phosphorylatable residues are also found in rice Lhcb1 and Lhcb2 (Figure 4B). As these Thr residues are preceded by a basic residue (Lys or Arg), they represent potential signatures recognized by STN7 [60]. In fact, the Thr-40 in Arabidopsis Lhcb2is phosphorylated in wild-type but not in the *stn7* mutant [59], further suggesting that it is a target of STN7. In D8QN27 (Lhcb1), we also identified the Ser-54 Pro-directed phosphosite which is conserved in Arabidopsis Lhcb1 [43] and an equivalent Ser residue in rice Lhcb1 (Figure 4B). Interestingly, all the N-terminal Ser/Thr residues mentioned above are not conserved in *P. patens* Lhcb1 and Lhcb2 and they were probably only evolved after the emergency of vascular plants. On the other hand, the Ser-48 and Ser-49 phosphosites in D8QN27 (Lhcb1) are located in a region not conserved with the Arabidopsis and rice sequences, but equivalent Ser residues are identified in moss Lhcb1. They may represent phosphorylation events that are lost in the angiosperm lineage.

Lhcb4, a minor chlorophyll-binding protein, was found to be phosphorylated in maize upon exposure to high light intensity for protection against cold stress [61]. The phosphosite Thr-112, a potential casein kinase II target, was identified in maize Lhcb4 [62]. This residue is not conserved in Selaginella Lhcb4 (D8RTB9) but present in Arabidopsis (pThr-109), rice (Thr-111), and moss (Thr-119) (Figure 4B). On the other hand, 2 consecutive phosphosites (Ser-57, 58) were detected in D8RTB9and the equivalent Ser residues are only found in moss but not in Arabidopsis or rice. While both of them are located in basic motifs, Ser-58 may also represent a target for acidic casein kinase II.

The PSII core proteins PsbA, PsbD and PsbC are also known to undergo a strong and dynamic redox-regulated phosphorylation cycle [63-65]. STN8-dependent phosphorylation of PSII proteins is required for rapid turn-over of photo-damaged PSII complexes and it is highly important during prolonged exposure of the photosynthetic apparatus to excess light [66]. As determined by its structure, STN8 kinase was reported to have a peculiar substrate specificity restricted to the very N-terminal Thr residue of PsbA, PsbD and PsbC [60]. For example, the phosphosite Thr-2 in Arabidopsis PsbD is phosphorylated by STN8 [42,58]. The same phosphorylation event is detected in Selaginella C7B2K2 (PsbD) while an equivalent Thr residue is found in rice (Figure 4B). On the other hand, while no N-terminal Thr phosphosites were identified in Selaginella C7B2K3 (PsbC), its Thr-346 phosphorylation is conserved in Arabidopsis PsbC [42,52] and equivalent Thr residues are found in rice and moss. This site may represent a substrate of acidic or basic motif recognizing kinases, indicating the possibility of cross-talk between kinases as suggested previously [60].

The oxygen-evolving complex (OEC) is consisted of PsbO, PsbP and PsbQ. PsbO stabilizes the manganese cluster which is the primary site of water splitting. Besides, PsbO regulates dephosphorylation and turnover of the PSII reaction center PsbA [67,68]. However, no phosphorylation events in PsbO have been reported previously in any plants. In Selaginella D8TBN9 (PsbO), we identified a unique Pro-directed Ser-219 phosphosite. The equivalent residues in other PsbO sequences examined are all Lys which is non-phosphorylatable (Figure 4B). PsbQ is required for PSII assembly, stability, and photoautotrophic growth under low light conditions [69]. The Selaginella PsbQ (D8S1M9) was found to be phosphorylated at the Ser-61 residue, which is a potential target of Pro-directed kinase. Equivalent pSer and Ser residues are found in Arabidopsis and moss PsbQ sequences, respectively (Figure 4B).

PsaC and PsaF are components of PSI which performs the light-induced electron transfer from plastocyanin or cytochrome c6 (Cytc) to ferredoxin. As a chloroplast-encoded PSI subunit, PsaC binds the two terminal electron acceptors (F_A_ and F_B_). No phosphorylation was reported in PsaC previously in any plants. PsaC is extremely conserved among the four plant species examined here with most of the residues identical (Additional file 6: Figure S2). Intriguingly, the phosphorylation event occurs at a unique residue (Ser-71) in Selaginella PsaC (C7B2J3). The equivalent residues in the other plant sequences are all non-phosphorylatable. The nuclear subunit PsaF provides a docking site for plastocyanin and Cytc on the lumenal side of PSI. In Arabidopsis, PsaF was reported to be phosphorylated at Ser-94, Ser-95, Tyr-99, and Ser-103 [42,52]. Most of the equivalent residues in Selaginella PsaF (D8QPQ3) are conserved except for Ser-95. On the other hand, the Ser-184 phosphosite in D8QPQ3 is located in the very C-terminal region which is absent in Arabidopsis and rice. The same residue was identified in the moss PsaF sequence, suggesting that the Ser-184 phosphorylation event might have been lost during the evolution of flowering plants.

Overall, several phosphorylated residues in the Selaginella photosynthesis proteins are conserved with equivalent phosphorylation in Arabidopsis and/or phosphorylatable residues in most of the plants examined, including Lhcb1: Thr-44, Lhcb2: Thr-42 and 46,PsbD: Thr-2,PsbC: Thr-346, and psbQ: Ser-61. The phosphorylation of Thr-46 in Lhcb2 is first identified in Selaginella and the equivalent residues in other plant sequences are likely to be phosphorylated. We also identified unique phosphorylated residues within highly conserved regions in Selaginella PsbO (Ser-219) and PsaC (Ser-71). On the other hand, phosphorylation events with equivalent residues only in moss were detected in Selaginella Lhcb1, Lhbc4 and PsaF. These phosphosites are located in low-homology regions when compared with the Arabidopsis and rice sequences, implicating that they were lost in the flowering plants during evolution. It will be very interesting to investigate how the different unique phosphorylation events are involved in light reactions in Selaginella.

## Conclusions

Our work generates the first large-scale atlas of phosphoproteins in Selaginella which occupies a unique position in the evolution of terrestrial plants. Combining PEG fractionation with IMAC enrichment, a total of 1593 unique phosphopeptides (1588 individual phosphosites) representing 851 unique phosphoproteins were retrieved. An overview of the Selaginella phosphoproteomics data revealed general features which are largely consistent with the dicot model Arabidopsis. Known plant phosphorylation Ser/Thr motifs were extracted from total and Selaginella-specific phosphopeptides, implicating the conservation of phosphorylation machineries during vascular plant evolution. In fact, 97highly conserved phosphorylation events were identified among Selaginella and flowering plant homologs. In PSI proteins, we identified conserved residues which are potential targets of STN7 and STN8 kinases. On the other hand, several phosphosites unique to Selaginella were detected in the highly conserved PSI and PSII proteins. Future research into functional roles of Selaginella-specific phosphorylation events in photosynthesis and other processes may offer insight into the molecular mechanisms leading to the distinct evolution of lycophytes.

## Methods

### Protein extraction and PEG fractionation

Two-gram aerial tissues of wild-growing *Selaginella moellendorffii* collected from the Victoria Peak in Hong Kong were ground to fine powder in liquid nitrogen. The powder was homogenized in 10 mL of ice-cold Mg/NP-40 extraction buffer containing 0.5 M Tris-HCl (pH 8.3), 20 mM MgCl_2_, 2% v/v NP-40, 2% v/v β-mercaptoethanol, 1 mM phenylmethylsulfonyl fluoride and 1% w/v polyvinylpolypyrrolidone using the Tissue-Tearor (BioSpec) operated at maximum speed for 1 min on ice [70]. After centrifugation at 12000 × g for 15 min at 4°C, the supernatant was treated with 15% PEG-4000 and incubated on ice for 30 min, followed by centrifugation at 1500 × g for 10 min at 4°C. The pellet was washed sequentially with ice-cold 10% trichloroacetic acid/acetone, ice-cold 100% methanol containing 0.1 M ammonium acetate, and ice-cold 100% acetone. The supernatant was precipitated by adding four volumes of ice-cold acetone and then incubated at -20°C for 2 h. After centrifugation at 12000 × g for 5 min at 4°C, the pellet was rinsed as described above. For the plant debris left after the initial Mg/NP-40 extraction, residual protein was extracted by 4% SDS. After centrifugation, the supernatant was precipitated with ice-cold acetone, followed by sequential rinsing of the pellet.

### Protein digestion and phosphopeptide enrichment

The pellets obtained from each of the above step were re-suspended in solution containing 0.2 M Tris-HCl (pH 8.0), 8 M urea and 4 mM CaCl_2_. Dissolved protein samples were reduced with 10 mM dithiothreitol for 30 min at 56°C, and the alkylated with 40 mM iodoacetamide for 30 min at room temperature in the dark. Protein concentration was measured by the Bio-Rad Protein Assay kit. Afterwards, trypsin (Worthington) was added in a 1:50 (enzyme: protein) w/w ratio and the mixture incubated overnight at 37°C. Trypsinized peptides were loaded onto a 1 g Sep-Pak C18 column (Waters), washed twice with 10 mL 1% acetic acid, eluted with 7 mL 80% acetonitrile containing 0.1% acetic acid, dried under speed-vacuum, re-suspended in 400 μL 1% acetic acid, and then loaded onto a mini-column of 40 μL IMAC resin prepared as described previously [71]. The IMAC mini-column was rinsed twice with 40 μL wash buffer containing 25% v/v acetonitrile, 100 mM NaCl and 0.1% v/v acetic acid, then washed once each with 40 μL 1% v/v acetic acid and 20 μL double-distilled water, eluted with 120 μL 6% w/v NH_3_.H_2_O, and dried under speed-vacuum. IMAC-enriched phosphopeptides derived from different PEG fractionated samples (Additional file 1: Figure S1) were subject to LC-MS/MS analysis.

### RPLC-ESI-MS/MS detection

The Triple TOF 5600 mass spectrometer (AB SCIEX), a hybrid quadrupole TOF platform [72], was coupled with an Nano-LC system (Agilent) utilizing Nanospray III ion-source (AB SCIEX). Mobile phase A (2% ACN, 0.1% formic acid) and mobile phase B (98% ACN, 0.1% formic acid) were used to establish a 120 min gradient comprised of 80 min (5-30% B), 12 min (30-60% B), 6 min (60-90% B), 10 min (90% B), and 12 min (90-5% B). The flow rate was 300 nL/min. Peptides were separated on a fused silica capillary emitter (New Objective) packed in-house with 5 μm C18 resin (New Objective), and analyzed in positive ion mode by electrospray ionization. For information dependent acquisition, each survey scan was acquired in 250 ms followed by 20 product ion scans collected in 50 ms/per scan.

### Database searching of MS/MS spectra

For proteome analysis, raw data from Triple TOF 5600 were searched with ProteinPilot software (version 4.0, AB SCIEX) against the Uniprot *Selaginella moellendorffii* complete proteome database (downloaded in April 2011, 33195 sequences) using following parameters: Sample Type (Identification), Cys Alkylation (Iodoacetamide), Digestion (Trypsin), Search Effort (Rapid). The false discovery rate (FDR) analysis was done by using the tool integrated in ProteinPilot. All data were filtered at 1% FDR.

For phosphoproteome analysis, raw data MS/MS (wiff files) were converted to .mgf files and searched with the Mascot (version 2.2, Matrix Science) software [73] against the Selaginella proteome database using following parameters: fixed modifications was set to carbamidomethylation on cysteine, variable modifications was set to oxidation of methionine and phosphorylation at serine, threonine and tyrosine, peptide and MS/MS fragment tolerances were set to 20 ppm and 0.2 Da respectively, trypsin was selected as digestion enzyme, and up to two missed cleavages were allowed. All .mgf files were merged into one file followed by database searching.

### Post-search data processing and phosphosite localization

The Mascot search result was first loaded into Scaffold (version 3.0, Proteome Software) for further analysis. In order to screen phosphopeptides with high confidence, “Min Protein” (protein identification probability), “Min # Peptide” (the number of unique peptides on which a protein identification is based) and “Min Peptide” (peptide identification probability) were adjusted to 20%, 1 and 95% respectively [74,75]. Afterwards, the mzIdentML file generated by Scaffold was loaded into Scaffold PTM (version 1.1, Proteome Software) to determine the localization probability of phosphosites [76].

### Gene ontology annotations

Gene ontology (GO) annotations of all identified proteins and phosphoproteins in Selaginella categorized into 3 classifications (Cellular Component, Molecular Function and Biological Process) were batch-retrieved from the Protein Information Resource (http://pir.georgetown.edu/pirwww/search/batch.shtml).

### Analysis of phosphorylation site conservation

The Selaginella phosphopeptides were clustered with different plant phosphopeptides retrieved from the Plant Protein Phosphorylation Database (P^3^DB; http://www.p3db.org/) using the CD-HIT web server [54] (http://www.bioinformatics.org/cd-hit/). All phosphopeptide sequences were combined into a single Fasta file for data upload. Default parameters were adopted together with a 60% similarity cutoff and a bandwidth of 5. Conservation of phosphorylation sites among different plant species were then identified by manual inspection of the sequence alignment in each cluster.

### Phosphorylation motif analysis

Sequence was centered on each phosphosite and extended to 15 amino acids (±7 residues). Phosphosites, which could not be extended because of N- or C-termini, were excluded from motif analysis. Only phosphosites with localization probability above 95% were used. General phosphorylation motif classes were assigned as defined previously [50]: P at +1 (Pro-directed); D/E at +1/+2 or +3 (Acidic), 5 or more D/E at +1 to +6 (Acidic); K/R at -3 (Basic), 2 or more K/R at -6 to -1 (Basic); otherwise (Others). Specific motifs were extracted from the data set by using motif-x algorithm (http://motif-x.med.harvard.edu/motif-x.html) [53]. The Selaginella proteome database in fasta format was retrieved (http://www.phytozome.com/) and uploaded as background. The significance threshold was set to 10^−6^ and the minimum number of motif occurrences was 20.

## Abbreviations

FDR: False discovery rate; GO: Gene ontology; IMAC: Immobilized metal affinity chromatography; P3DB: Plant protein phosphorylation database; PEG: Polyethylene glycol; PSI: Photosystem I; PSII: Photosystem II; PTMs: Post-translational modifications; RUBISCO: Ribulose-1,5-bisphosphate carboxylase/oxygenase.

## Competing interests

The authors declare that they have no competing interests.

## Authors’ contributions

XC, WLC, and CL initially conceived of and designed the proteomics experiments. XC and WLC participated in all experimental procedures, data analysis, and manuscript preparation. FYZ was involved in data analysis. CL finalized the manuscript for submission. All authors read and approved the final manuscript.

## Supplementary Material

Additional file 1: Figure S1 SDS-PAGE analysis of protein samples after PEG fractionation. Total protein (T) was obtained from the supernatant after extraction of tissues in Mg/NP-buffer. Residual protein (R) was obtained by extracting the plant debris in 4% SDS. The supernatant was subject to 15% PEG precipitation and the pellet (P) was resuspended in urea-containing buffer. Protein in the final supernatant (S) was precipitated by acetone and resuspended in urea-containing buffer. Each lane was loaded with 50 μg of protein. Following Coomassie blue staining, the large subunit of RUBISCO (RBCL) can be visualized as a discrete band corresponding to a MW of ~55 kDa. Note the substantially reduced abundance of RBCL in the sample (S) after PEG precipitation. The fractionated samples (S, P, and R) were trypsin-digested, followed by IMAC enrichment of phosphopeptides. Click here for file

Additional file 2: Table S1 List of all identified Selaginella phosphopeptides. Click here for file

Additional file 3: Table S2 List of 716 Selaginella phosphoproteins (localization probability ≥ 95%) with OrthoMCL Group information. Click here for file

Additional file 4: Table S3 List of Selaginella phosphopeptides with the KXXS motif. Click here for file

Additional file 5: Table S4 List of Selaginella phosphopeptides with conserved phosphosites in other plant phosphopeptides. Click here for file

Additional file 6: Figure S2 Complete ClustalW sequence alignment of the nine identified Selaginella PSI proteins with Arabidopsis, rice and moss orthologous sequences. Phosphosites identified in this study and in Arabidopsis are highlighted in red and yellow, respectively. The rice and moss sequences are included as references although no phosphorylation information is available for these proteins. Click here for file
