# Supplementary material for: Phosphoproteomic analysis of the non-seed vascular plant model Selaginella moellendorffii
Source: Proteome Sci. 2014 Mar 17;12:16. doi: 10.1186/1477-5956-12-16 (PMC4022089; doi:10.1186/1477-5956-12-16)
Supplement: Additional file 1: Figure S1 — SDS-PAGE analysis of protein samples after PEG fractionation. Total protein (T) was obtained from the supernatant after extraction of tissues in Mg/NP-buffer. Residual protein (R) was obtained by extracting the plant debris in 4% SDS. The supernatant was subject to 15% PEG precipitation and the pellet (P) was resuspended in urea-containing buffer. Protein in the final supernatant (S) was precipitated by acetone and resuspended in urea-containing buffer. Each lane was loaded with 50 μg of protein. Following Coomassie blue staining, the large subunit of RUBISCO (RBCL) can be visualized as a discrete band corresponding to a MW of ~55 kDa. Note the substantially reduced abundance of RBCL in the sample (S) after PEG precipitation. The fractionated samples (S, P, and R) were trypsin-digested, followed by IMAC enrichment of phosphopeptides. [file 1477-5956-12-16-S1.pdf]

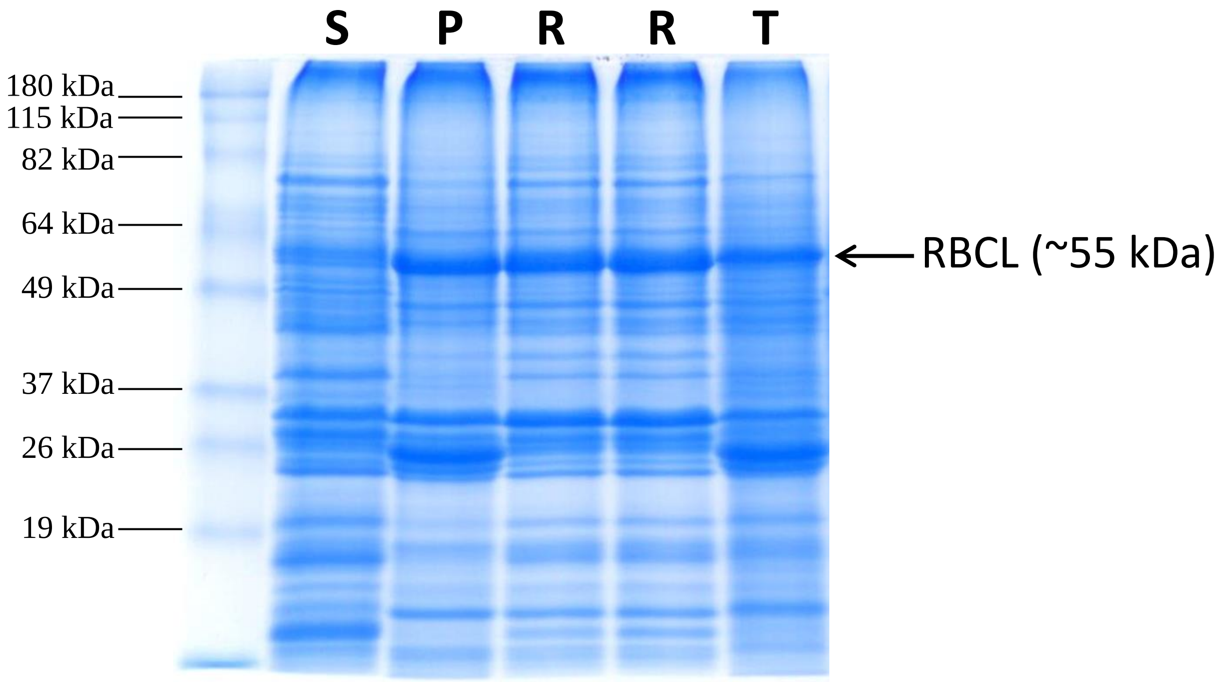

Figure S1. SDS-PAGE analysis of protein samples after PEG fractionation. Total protein (T) was obtained from the supernatant after grinding of plant tissues in Mg/NP-buffer. The plant debris was extracted in 4% SDS to obtain the residual protein (R). The total protein (T) sample was subject to 15% PEG precipitation and the pellet (P) was re-suspended in urea-containing buffer. Protein in the final supernatant (S) was precipitated by acetone and re-suspended in urea-containing buffer. For SDS-PAGE analysis, each lane was loaded with 50 μg of protein. Following Coomassie blue staining, the large subunit of RUBISCO (RBCL) can be visualized as a discrete band corresponding to a MW of ~55 kDa. Note the substantially reduced abundance of RBCL in the sample (S) after PEG precipitation. The fractionated samples (S, P, and R) were then trypsin-digested, followed by IMAC enrichment of phosphopeptides.
