# Supplementary material for: Phosphoproteomic analysis of the non-seed vascular plant model Selaginella moellendorffii
Source: Proteome Sci. 2014 Mar 17;12:16. doi: 10.1186/1477-5956-12-16 (PMC4022089; doi:10.1186/1477-5956-12-16)
Supplement: Additional file 6: Figure S2 — Complete ClustalW sequence alignment of the nine identified Selaginella PSI proteins with Arabidopsis, rice and moss orthologous sequences. Phosphosites identified in this study and in Arabidopsis are highlighted in red and yellow, respectively. The rice and moss sequences are included as references although no phosphorylation information is available for these proteins. [file 1477-5956-12-16-S6.pdf]

Lhcb1

D8QN27_SELML:
AT1G29930.1:
Os01g41710.1_ORYSA:
A9S6S7_PHYPA:

```
MAAMTATATMAAPSTFAGQTGLKPQNEFVARNASNGSRIAMRKtG-KAss 49
MAASTMALS---SPAFAGKAVKLsP---AAsEVLGSGRVTMRKtVAKPK- 43
MAAATMALS---SPALAGK---------AAAKVFGEGRITMRKSAAKPKP 38
MAACTATHS----TALAGQTALKSINELSRKVGNVQARVTMRK-ASSSDS 45
*** * : :    .::**:              .*::*** .....

VGGGsPWYGPDRVKYLGPFSGESPSYLTGEYPGDYGWDTAGLSADPETFA 99
GPSGsPWYGSDRVKYLGPFSGESPSYLTGEFPGDYGWDTAGLSADPETFA 93
AASGSPWYGADRVLYLGPLSGEPPSYLTGEFPGDYGWDTAGLSADPETFA 88
I-----WYGADRPKYLGPLSGETPSYLTGEFAGDYGWDTAGLSSDPETFA 90
***.**  ****:***.*******:.***********:******

KNRELEVIHSRWAMLGALGCVFPELLSKNGIKFGEAVWFKAGSQIFKEGG 149
RNRELEVIHSRWAMLGALGCVFPELLARNGVKFGEAVWFKAGSQIFSDGG 143
KNRELEVIHSRWAMLGALGCVFPELLARNGVKFGEAVWFKAGSQIFSEGG 138
RNRELEVIHARWAMLGALGCVTPELLAKNGVKFGEAVWFKAGSQIFSEGG 140
:********:*********** ****::**:***************.:**

LDYLGNPSLVHAQSILAIWASQVILMGAVEGYRIAG-GPLGEVTDPIYPG 198
LDYLGNPSLVHAQSILAIWATQVILMGAVEGYRVAGNGPLGEAEDLLYPG 193
LDYLGNPSLIHAQSILAIWAVQVVLMGAVEGYRIAG-GPLGEVVDPLYPG 187
LDYLGNPSLVHAQSILAIWASQVILMGAVEGYRVAG-GPLGEVTDPIYPG 189
*********:********** **:*********:** *****. * :***

GSFDPLGLAEDPEAFAELKVKELKNGRLAMFSMFGFFVQAIVTGKGPLEN 248
GSFDPLGLATDPEAFAELKVKELKNGRLAMFSMFGFFVQAIVTGKGPIEN 243
GSFDPLGLADDPEAFAELKVKEIKNGRLAMFSMFGFFVQAIVTGKGPLEN 237
GSFDPLGLADDPDTFAELKVKEIKNGRLAMFSMFGFFVQAIVTGKGPLEN 239
********* **::********:************************:**

LSDHLADPVANNAWAYATNFTPGS 272
LADHLADPVNNNAWAFATNFVPGK 267
LADHLADPVNNNAWAYATNFVPGK 261
LSDHLADPVANNAWAYATNFVPGN 263
*:******* *****:****.**.
```

Lhcb2

D8SUF1_SELML:
AT2G05100.1:
Os03g39610.1_ORYSA:
A9S6S7_PHYPA:

```
MAASASAISSSSFSGQTALKSPNELSRKVGNLGEARTVMRRtRAStPDSI 50
-MATSAIQQS-SFAGQTALKPSNELLRKVGVSGGGRVTMRRtVKSTPQSI 48
-MAASALHQTTSFLGTAPRR--DELVRRVGDSGG-RITMRRTVKSAPQSI 46
-MAACTATHSTALAGQTALKSINELSRKVGNV-QARVTMR--KASSSDSI 46
*:.:  : :: * :. :  :** *:**    * .**   *:.:**

WYGPDRPKFLGPFSEQTPSYLTGEFPGDYGWDTAGLSADPETFAKNRELE 100
WYGPDRPKYLGPFSENTPSYLTGEYPGDYGWDTAGLSADPETFAKNRELE 98
WYGPDRPKYLGPFSEQTPSYLTGEFPGDYGWDTAGLSADPETFARNRELE 96
WYGADRPKYLGPLSGETPSYLTGEFAGDYGWDTAGLSSDPETFARNRELE 96
***.****:***:* :********:.***********:******:*****

VIHSRWAMLGALGCVTPELLAKNGIKFGEAVWFKAGSQIFKEGGLDYLGN 150
VIHSRWAMLGALGCTFPEILSKNGVKFGEAVWFKAGSQIFSEGGLDYLGN 148
VIHSRWAMLGALGCVFPEILSKNGVKFGEAVWFKAGAQIFSEGGLDYLGN 146
VIHARWAMLGALGCVTPELLAKNGVKFGEAVWFKAGSQIFSEGGLDYLGN 146
***:**********. **:*:***:***********:***.*********

PNLVHAQSILAIWASQVILMGAVEGYRVGGGPLGEGLDKIYPGGAFDPLG 200
PNLIHAQSILAIWAVQVVLMGFIEGYRIGGGPLGEGLDPLYPGGAFDPLN 198
PNLVHAQSILAIWAVQVVLMGFVEGYRVGGGPLGEGLDKVYPGGAFDPLG 196
PSLVHAQSILAIWASQVILMGAVEGYRVAGGPLGEVTDPIYPGGSFDPLG 196
*.*:********** **:*** :****:.******  * :****:****.

LADDPDTFAELKVKELKNGRLAMFSMFGFFTQAIVTGKGPIENLLDHLDN 250
LAEDPEAFSELKVKELKNGRLAMFSMFGFFVQAIVTGKGPIENLFDHLAD 248
LADDPDTFAELKVKELKNGRLAMFSMFGFFVQAIVTGKGPIENLFDHVAD 246
LADDPDTFAELKVKEIKNGRLAMFSMFGFFVQAIVTGKGPLENLSDHLAD 246
**:**::*:******:**************.*********:*** **: :

PTVNNAWAYATNFTPGS 267
PVANNAWSYATNFVPGN 265
PVANNAWAYATNFVPGK 263
PVANNAWAYATNFVPGN 263
*..****:*****.**.
```

Lhcb4

D8RTB9_SELML:
AT3G08940.2:
Os07g37240.1_ORYSA:
A9U3M1_PHYPA:

```
-MASALAASSFTGCGVRDLVGYAAASSTSQVASRSSGIVARFGLGKKKGT 49
MAATSTAAAASSIMGTRV---------VSDISSNSSRFTARFGFG-TKKA 40
-MASSVAAAASTFLGTR----------LADPAPQSGRIVARFGFGGGKKA 39
-MAQALRSASIAASSFVG-------SVECFGVKNGARVVARSSLGGKAKQ 42
* :  ::: : .           .    ... ..** .:*

KK----VTKKSSSGDANRPLWFPGAKCPDYLDGSLVGDYGFDPLGLGKP 95
SP----KKAK---TVISDRPLWFPGAKSPEYLDGSLVGDYGFDPFGLGKP 83
AA----KKAARPSAPTTDRPLWFPGAVAPDYLDGSLVGDYGFDPFGLGKP 85
VAKQASKTLKKSSGAGADRPLWFPGARAPEWLDGSLPGDYGFDPLSLGKS 92
.       ::******** .*::***** *******:.***.

PTYLQFDVDSLDSSLAKNMPGELLGERVEDLSTIRATPLQPYTEIFGLQR 145
AEYLQFDLDSLDQNLAKNLyGEVIGtRtEAVD-PKSTPFQPYSEVFGLQR 132
AEYLQFELDSLDQNLAKNNAGEIIGTRFETGE-VKSTPFQPYTEVFGLQR 134
PEYLQIELDSLNQNLAKNEAGDVIGTRISKKEEIAPTPFQPYSEVFGLQR 142
. ***:::***:..****  *:::* * .  .   .**:***:*:*****

FRECELIHGRWAMLATLGAIAVETFTGVTWQDAGKVELDQGPSYFGFSLP 195
FRECELIHGRWAMLATLGAITVEWLTGVTWQDAGKVELVDGSSYLGQPLP 182
FRECELIHGRWAMLATLGALSVEWLTGVTWQDAGKVELVDGSSYLGQPLP 184
FRECELIHGRWAMLAILGALSVEAFTGVTWQDAGKVELVDGASYFGLPLP 192
*************** ***::** :************* :*.**:* .**

FTITALVWIEVLVIGFIEFQRNTELDPEKRLYPGGSYFDPLGLAADPERK 245
FSISTLIWIEVLVIGYIEFQRNAELDSEKRLYPGGKFFDPLGLASDPVKK 232
FSISTLIWIEVLVIGYIEFQRNAELDPEKRLYPGGSYFDPLGLASDPEKK 234
FSITSLVYIEAFLVGFIEFQRNAELDPEKRLYPGGKFFDPLNFADTEEKK 242
*:*::*::**.:::*:******:***.********.:****.:*   :*

EVLKLAEIKHARLAMVAAFGFAVQAAATGKGPLDNWVTHLSDPLHTTIID 295
AQLQLAEIKHARLAMVGFLGFAVQAAATGKGPLNNWATHLSDPLHTTIID 282
ERLQLAEIKHARLAMVAFLGFAVQAAATGKGPLNNWATHLSDPLHTTIFD 284
ETLKLAEIKHARLAMVAALGFAAQAAATGKGPLDNWATHLADPLHTTIFD 292
*:************. :***.**********:**.***:*******:*
```

TFSK-- 299
TFSSS- 287
TFSSSS 290
TFSK-- 296
***.

PsbD

C7B2K2_SELML:
ATCG00270.1:
Os02g24634.1_ORYSA:

```
MtIVVGRLSGGSTSFFDNTDDRPRRDRFAFVGRSGLSLLPRAYFSPGGWF 50
MtIALGKFTKDEKDLFDIMDDWLRRDRFVFVGWSGLLLFPCAYFALGGWF 50
MTIALGRVTKEENDLFDIMDDWLRRDRFVFVGWSGLLLFPCAYFALGGWF 50
***.:*:.:  ...:**  **  *****.*** *** *:* ***: ****

TGTTSVTSRYTHGLASSYPEGRNSLTVAASTPANSPAHSPPLLWGPEAQG 100
TGTTFVTSWYTHGLASSYLEGCNFLTAAVSTPANSLAHSLLLLWGPEAQG 100
TGTTFVTSWYTHGLASSYLEGCNFLTAAVSTPANSLAHSLLLLWGPEAQG 100
**** *** ********* ** * **.*.****** ***  *********

DFTRWCQLGGLWTFVALHGAFGLMGPMLRQSELARSVQSRPYNAIAFSAP 150
DFTRWCQLGGLWAFVALHGAFALIGFMLRQFELARSVQLRPYNAIAFSGP 150
DFTRWCQLGGLWTFVALHGAFALIGFMLRQFELARSIQFRPYNAISFSGP 150
************:********.*:* **** *****:* ******:**.*

IAVSVPVSLIHPLGQSGRFSAPSSGVAAIFRSILLPQGLHNWTLNPFHMM 200
IAVFVSVFLIYPLGQSGWFFAPSFGVAAIFRFILFFQGFHNWTLNPFHMM 200
IAVFVSVFLIYPLGQSGWFFAPSFGVAAIFRFILFFQGFHNWTLNPFHMM 200
*** *.* **:****** * *** ******* **: **:***********

GVAGVPGAAPPRATHGATVENTLFEDGDGANTFRASNPTQSEETYSMVTA 250
GVAGVLGAALLCAIHGATVENTLFEDGDGANTFRAFNPTQAEETYSMVTA 250
GVAGVLGAALLCAILGATVENTLFEDGDGANTFRAFNPTQAEETYSMVTA 250
***** ***   *  ******************** ****:*********

NRFWSQTSGVAPPNKRRSHFPMLFVPVTGSRMSAIGVVGLAPNLRAYDFV 300
NRFWSQIFGVAFSNKRWLHFFMLFVPVTGLWMSALGVVGLALNLRAYDFV 300
NRFWSQIFGVAFSNKRWLHFFMLFVPVTGLWMSAIGVVGLALNLRAYDFV 300
******  *** .***  ** ********  ***:****** ********

SQEVRAAEDPEFETPYTKNILSNEGIRAWMAAQDQPHENLVFPEEVLPRG 350
SQEIRAAEDPEFETFYTKNILLNEGIRAWMAAQDQPHENLIFPEEVLPRG 350
SQEIRAAEDPEFETFYTKNILLNEGIRAWMAAQDQPHENLIFPEEVLPRG 350
***:********** ****** ******************:*********
```

NAL 353
NAL 353
NAL 353
***

PsbC

C7B2K3_SELML:
ATCG00280.1:
Os02g24632.1_ORYSA:
PSBC_PHYPA:

```
MKTSYSPRRFYPVETLFDGTLALGGRDQETTGFAWWAGNARLINLSGKSL 50
MKTLYSLRRFYHVEtLFNGTLALAGRDQETTGFAWWAGNARLINLSGKLL 50
MKILYSLRRFYHVETLFNGTFVLAGRDQETTGFAWWAGNARLINLSGKLL 50
MKILYSQRRFYHVETLFNGTLALSGRDQETTGFAWWAGNARLINLSGKLL 50
**  ** **** *****:**:.*.************************ *

GAHVAHAGLIVFWAGAMTLFEVAHFVPEKPMYEQGLILLPHLATLGWGVG 100
GAHVAHAGLIVFWAGAMNLFEVAHFVPEKPMYEQGLILLPHLATLGWGVG 100
GAHVAHAGLIVFWAGAMNLFEVAHFVPEKPMYEQGLILLPHLATLGWGVG 100
GAHVAHAGLIVFWAGAMNLFEVAHFVPEKPMYEQGLILLPHLATLGWGVG 100
*****************.********************************

PGGEAVGVSPYLASGVLHLISSAVLGFGGIYHSLIGPDTLEESFPFFGYE 150
PGGEVIDTFPYFVSGVLHLISSAVLGFGGIYHALLGPETLEESFPFFGYV 150
PGGEVLDTFPYFVSGVLHLISSAVLGFGGIYHALLGPETLEESFPFFGYV 150
PGGEVIDTFPYFVSGVLHLISSAVLGFGGIYHALIGPETLEESFPFFGYV 150
****.:.. **:.*******************:*:**:***********

WRNRNKMTTILGIHLILLGIGALLLVSKALYFGGVYDTWAPGGGDVREIT 200
WKDRNKMTTILGIHLILLGVGAFLLVFKALYFGGVYDTWAPGGGDVRKIT 200
WKDRNKMTTILGIHLILLGIGAFLLVLKALYFGGIYDTWAPGGGDVRKIT 200
WKDKNKMTTILGIHLILLGAGAFLLVFKALYFGGIYDTWAPGGGDVRKIT 200
*:::*************** **:*** *******:************:**

NLTASPSVISGYLLKSPFGGEGWIVSVDNLEDVIGGHLWLGSICILGGIF 250
NLTLSPSVIFGYLLKSPFGGEGWIVSVDDLEDIIGGHVWLGSICIFGGIW 250
NLTLSPGVIFGYLLKSPFGGEGWIVSVDDLEDIIGGHVWLGFICVFGGIW 250
NLTLSPGVIFGYLLKSPFGGEGWIVSVDNLEDIIGGHVWLGSICIFGGIW 250
*** **.** ******************:***:****:*** **::***:

HILTKPFAWARRAFVWSGEAYLSYSLGALSIFGFIACCFVWFNNTVYPSE 300
HILTKPFAWARRALVWSGEAYLSYSLAALSVCGFIACCFVWFNNTAYPSE 300
HILTKPFAWARRAFVWSGEAYLSYSLGALSVFGFIACCFVWFNNTAYPSE 300
HILTKPFAWARRALVWSGEAYLSYSLGAIAVFGFIACCFVWFNNTAYPSE 300
*************:************.*::: *************.****
```

```
FYGPTGPEASQAQAFTFLVRDQRLGANAGSAQGPTGLGKYLMRSPtGEII 350
FYGPTGPEASQAQAFTFLVRDQRLGANVGSAQGPTGLGKYLMRsPtGEVI 350
FYGPTGPEASQAQAFTFLVRDQRLGANVGSAQGPTGLGKYLMRSPTGEVI 350
FYGPTGPEASQAQAFTFLVRDQRLGANVGSAQGPTGLGKYLMRSPTGEII 350
***************************.********************:*

FGGETMRFWDLRAPWLEPLRGPNGLDLSKLKRDIQPWQERRSAEYMTHAP 400
FGGEtMRFWDLRAPWLEPLRGPNGLDLSRLKKDIQPWQERRSAEYMTHAP 400
FGGETMHFWDLRAPWLEPLRGPNGLDLSRLKKDIQPWQERRSAEYMTHAP 400
FGGETMRFWDLRAPWLEPLRGPNGLDLSKLKKDIQPWQERRSAEYMTHAP 400
******:*********************:**:******************

LGSLNSVGGVATEINAVNYVSPRSWLATSHFVLGFFFFVGHLWHAGRARA 450
LGSLNSVGGVATEINAVNYVSPRSWLSTSHFVLGFFLFVGHLWHAGRARA 450
LGSLNSVGGVATEINAVNYVSPRSWLATSHFVLGFFFFVGHLWHAGRARA 450
LGSLNSVGGVATEINAVNYVSPRSWLATSHFVLGFFFFVGHLWHAGRARA 450
**************************:*********:*************

AAAGFERGIDRDSEPVLSMTPLN 473
AAAGFEKGIDRDFEPVLsMTPLN 473
AAAGFEKGIDRDLEPVLYMTPLN 473
AAAGFEKGIDRDFEPVLSMTPLN 473
******:***** **** *****
```

PsbO

D8TBN9_SELML:
AT3G50820.1:
Os01g31690.1_ORYSA:
A9RS28_PHYPA:

```
MAASVVAAKAGPSLLQPKLDASAGSSASLRPVSVSKAFGLRSNGGGARLT 50
MATSLQAAATFLQPAKIAASPS-RNVHLRSNQTVGKSFGLDS--SQARLT 47
MAASLQAAATLMQPAKLGGRASSAALPSRPSSHVARAFGVDTG-AAGRIT 49
MAASVASVGCKVTAALAS-SAKVEKIQAVRSVSMSNAFGLKP--AVARVS 47
**:*: :.            ..           :..:**: .  . .*::

CSMEDIVKRAT----DVGKVAVATAAATLLVAGTAAAN--PKRLTYDEVQ 94
CSLHSDLKDFAGKCSDAAKIAGFALATSALVVSGAGAEGAPKRLTYDEIQ 97
CSLQSDIREVANKCADAAKLAGFALATSALLVSGASAEGVPRRLTFDEIQ 99
CNLEENVKSIV----DNAKAAAIVLASSALVAGAAFAEGVPQRLTFDEVN 93
*.:.. ::  .    * .* *  . *:: *:.. * *:  *:***:**::

AQTYLEVKGSGTANQCPILSGGDEGFKFKPGSYSVKKFCLEPTSFTVKKE 144
SKTYMEVKGTGTANQCPTIDGGSETFSFKAGKYTGKKFCFEPTSFTVKAD 147
SKTYMEVKGTGTANQCPTVEGGVDSFAFKAGKYNMKKFCLEPTSFTVKAE 149
AQTYMDVKGSGTANQCPIVAEGSDKFAFKDGNYTMRKFCLEPTSFTVKAE 143
::**::***:******* :  * : * ** *.*. :***:******** :

SPFKGGGDQYVDTKLMTRLTYTLDEIEADLSVDEKGNLKLVEKDGIDYAA 194
SVSKNAPPDFQNTKLMTRLTYTLDEIEGPFEVGSDGSVKFKEEDGIDYAA 197
GVAKNAPPEFQKTKLMTRLTYTLDEIEGPLEVSSDGTIKFEEKDGIDYAA 199
SQFKGGNEGFQPTKLMTRLTYTLDEIEGPLTVQ-NGNLRFEEVDGIDYAA 192
.  *..   :  ***************. : *  .*.::: * *******

VTVQLPGGERVPFLFTIKELVATGsPDGFSGSFLVPSYRGSSFLDPKGRG 244
VTVQLPGGERVPFLFTVKQLEASGKPESFSGKFLVPSYRGSSFLDPKGRG 247
VTVQLPGGERVPFLFTIKNLVATGKPESFGGPFLVPSYRGSSFLDPKGRG 249
VTVQLPGGERVPFLFTVKELVAEGKPEAFGGSFLVPSYRGSSFLDPKGRG 242
****************:*:* * *.*:.*.* ******************

GSTGYDNAVALPAGGAGDEEELGKENLKDTSGSTGNITLKVAESNTATGE 294
GSTGYDNAVALPAGGRGDEEELSKENVKNTAASVGEITLKITKSKPETGE 297
GSTGYDNAVALPAGGRGDEEELAKENVKNASSSTGNITLSVTKSKPETGE 299
GSTGYDNAVALPAGGAGDEEELAKENEKNTAALNGNITFSIAKSNVQTGE 292
*************** ******.*** *:::.  *:**:.:::*:  ***
```

```
IAGVFESIQPSDTDLGSKAPKEVKIQGIWYAQLD 328
VIGVFESLQPSDTDLGAKVPKDVKIQGVWYGQIE 331
VIGVFESVQPSDTDLGAKVPKDVKIQGVWYAQLE 333
IAGTFESFQPSDTDLGSKAPKEVKIEGIFYAQLD 326
: *.***.********:*.**:***:*::*.*::
```

PsbQ

D8S1M9_SELML:
AT4G05180.1:
Os07g36080.3_ORYSA:
A9S1E8_PHYPA:

```
MVGLVAVAAGVLAVEES--------------------------------- 17
MAQAVTSMAGLRGASQAVLEGSLQING---SNRLNISRVSVGSQRTGLVI 47
MAQAALAEA----------------------------------------- 9
MAN-TVAMAGLCGTSQALTQGGLVSCNNVINGSSRVNSVCVSKPAVVVAR 49
*.  .  *

----------------------------------RALTGIKINGPPPPSG 33
RAQQNVSVPESSRRSVIGLVAAGLAGGSFVKAVFAEAIPIKVGGPPLPSG 97
------------------------------------AKPIKLGPPPPPSG 23
AEQSDAAASVQSRRSVLSLLAATIVGTAVVNEARADARSVKLEPPPPLSG 99
:*:  **  **

GLPGTENADQPRDLDLPLKERFFIQPLsPAEAVGRIKDASKDIVGVKELI 83
GLPGTDNSDQARDFSLALKDRFyIQPLsPtEAAARAKDSAKEIINVKSFI 147
GLPGTLNSDQARDTDLPLRERFYLQPLPPAEAAARAKESAQDIINLKPLI 73
GLPGTENADQARDTDLPLRERFFIQPLSPADAAQRAKFSAQDIINVKSLI 149
***** *:**.** .*.*::**::***.*::*. * * ::::*:.:* :*

DKKSWPYVRNDLRNKATYLRYDLKTIMDAKPKAERKALKKLTDNLFEVID 133
DKKAWPYVQNDLRLRASYLRYDLNTVISAKPKEEKQSLKDLTAKLFQTID 197
EKKQWPFVRDDLRLRASYLRYDLKTVINSKPKDEKKGLKDLTGKLFATID 123
DKKAWPYVQNGLRSSAGYLRYDLNTVITSKSKEDRKSLKALSLKLFESLN 199
:** **:*::.**  * ******:*:: :*.* :::.** *: :**  ::

KLDFAARAKNPTDAGKCYAEAVAALDTVIAKISA 167
NLDYAARSKSSPDAEKYYSETVSSLNNVLAKLG- 230
GLDHAAKIKSPEEAEKYYTLTKSALGDVLAKLG- 156
ALDYAARSKSTKDAEKYYSQTVTLLNDVLSKIA- 232
**.**: *.. :* * *: : : *. *::*:.
```

PsaC

C7B2J3_SELML:
ATCG01060.1:
Os10g21406.1_ORYSA:
PSAC_PHYPA:

```
MAHPAKIHGTCIGRTQCVRACPTDVSETIPWDGCKANQIASAPRTEDRVG 50
MSHSVKIYDTCIGCTQCVRACPTDVLEMIPWDGCKAKQIASAPRTEDCVG 50
MSHSVKIYDTCIGCTQCVRACPTDVLEMIPWDGCKAKQIASAPRTEDCVG 50
MAHSVKIYDTCIGCTQCVRACPTDVLEMVPWDGCKASQIASAPRTEDCVG 50
*:*..**:.**** *********** * :*******.********** **

CKRRESACPTDPSSARVYLGSETTRSTGLAHQ 82
CKRCESACPTDFLSVRVYLWHETTRSMGLAY- 81
CKRCESACPTDFLSVRVYLGPETTRSMALSY- 81
CKRCESACPTDFLSVRVYLGAETTRSMGLAY- 81
*** *******  *.****  ***** .*::
```

PsaF

D8QPQ3_SELML:
AT1G31330.1:
Os03g56670.1_ORYSA:
A9TWD4_PHYPA:

```
--------------------------------------------------
MSLTIPANLVLNPRSNKSLTQSVPKSSARFVCSDD-------KSSSSTPQ 43
MAALAAASTAFAAKPRLARAPSSPAARFSVSCSASGNNGGAGEMAQSLAA 50
--------------------MFVKNTKARTVCSAS------ADETATVAQ 24

----MAAALAAAAIIGSAPIVAAPPEAAADVAGLTKCKDSAAFAKREKKE 46
SMKAFSAAVALSSILLS-----APMPAVADISGLTPCKDSKQFAKREKQQ 88
SAKTFSAALALSSVLLSS-AATSPPPAAADIAGLTPCKESKAFAKREKQS 99
TAGKFATALALAAIVGGSDMVVP--EARADVAGLTPCKESKGFAKRQKQE 72
:::*:* :::: .     .   * **::*** **:*  ****:*:.

IKKLQSRLKLYADDSAPALAINATIEKTKRRFKFYGDAGLLCGADGLPHL 96
IKKLEssLKLyAPEsAPALALNAQIEKTKRRFDNYGKYGLLCGSDGLPHL 138
IKKLQSSLKKYAPDSAPALAINATIEKTKRRFENYGKFGLLCGADGLPHL 149
IKKLESRLKLYAPDSAPALAINATIEKTKRRFEFYGKQGLLCGTDGLPHL 122
****:* ** ** :******:** ********. **. *****:******

IVDGDQQHLGEFVYPGLIFLYIAGWIGWVGRSYLIAVSTEAKPTQKEIII 146
IVNGDQRHWGEFITPGILFLYIAGWIGWVGRSYLIAISGEKKPAMKEIII 188
IVSGDQRHWGEFITPGLLFLYIAGWIGWVGRSYLIAISGEKKPAMREIII 199
IVDGDQAHLGEFVYPGLVFLYIAGWIGWVGRAYLIDVRTSKKPTEKEIII 172
**.*** * ***: **::*************:*** :  . **: :****

DVPLATSLIWKGFVWPLAAVSEFRNGKLVVDAGNITVsPR 186
DVPLASRIIFRGFIWPVAAYREFLNGDLIAKDV------- 221
DVELAVKLLPRGFIWPVAAYRELITGNLVVDDADIGY--- 236
DVPLALRVMSKGFTWPLAAIGELRSGKLVEKSGNITVSPR 212
** **  :: :** **:**  *: .*.*: .
```

Supplementary Figure S2. Complete ClustalW sequence alignment of Selaginella photosynthesis proteins with Arabidopsis, rice and moss orthologs. Phosphosites identified by our group and other groups are highlighted in red and yellow, respectively. The moss sequences are included as a reference although no phosphorylation information is available.
